# Identifying clinical subtypes in acute dyspnoea patients admitted to the emergency department: a secondary model-based clustering of prospective cohorts

**DOI:** 10.1093/eschf/xvag225

**Published:** 2026-08-25

**Authors:** Kamilė Čerlinskaitė-Bajorė, Nikola Kozhuharov, Mojtaba Ahmadiankalati, Desiree Wussler, Maria Belkin, Christian Mueller, Jelena Čelutkienė, Alexandre Mebazaa, Sabri Soussi

**Affiliations:** Clinic of Cardiac and Vascular Diseases, Institute of Clinical Medicine, Faculty of Medicine, Vilnius University, Vilnius, Lithuania; Cardiovascular Research Institute Basel (CRIB) and Department of Cardiology, University Hospital Basel, University of Basel, Basel, Switzerland; Department of Cardiology, Inselspital, Bern University Hospital, University of Bern, Bern, Switzerland; Department of Anesthesiology Research, St. Paul’s Hospital, Providence Health Care, Vancouver, BC, Canada; Cardiovascular Research Institute Basel (CRIB) and Department of Cardiology, University Hospital Basel, University of Basel, Basel, Switzerland; Department of Cardiology, Vancouver General Hospital, Vancouver, BC, Canada; Cardiovascular Research Institute Basel (CRIB) and Department of Cardiology, University Hospital Basel, University of Basel, Basel, Switzerland; Department of Hematology, University Hospital Basel, Basel, Switzerland; Cardiovascular Research Institute Basel (CRIB) and Department of Cardiology, University Hospital Basel, University of Basel, Basel, Switzerland; Clinic of Cardiac and Vascular Diseases, Institute of Clinical Medicine, Faculty of Medicine, Vilnius University, Vilnius, Lithuania; Department of Anesthesiology, Critical Care, Lariboisière-Saint-Louis Hospitals, DMU Parabol, AP-HP Nord, University of Paris, Paris, France; Inserm UMR-S 942, Cardiovascular Markers in Stress Conditions (MASCOT), Paris, France; Shock and Acute Conditions Outcomes Platform Consortium (ShockCO-OP), University of Toronto, Toronto, ON, Canada; Shock and Acute Conditions Outcomes Platform Consortium (ShockCO-OP), University of Toronto, Toronto, ON, Canada; Department of Anesthesia and Pain Management, University Health Network (UHN), University of Toronto, Toronto Western Hospital, 399 Bathurst Street, Toronto, ON, Canada M5T 2S8

**Keywords:** Acute dyspnoea, Clinical subtypes, Emergency department, Risk stratification, Latent class analysis

## Abstract

**Introduction:**

Acute dyspnoea is a frequent reason for emergency department (ED) presentation, with heterogeneous causes and prognosis often insufficiently captured by traditional diagnostic categories. We applied model-based clustering (i.e. latent class analysis [LCA]) to identify distinct subtypes of acute dyspnoea and assess their prognostic relevance.

**Methods:**

We analysed two prospective ED acute dyspnoea adult cohorts (LEDA and BASEL V). LCA was performed in the LEDA derivation cohort using nine admission clinical/biological variables. A simplified decision tree was derived in the LEDA cohort and externally validated to classify BASEL V patients. Subtypes were compared regarding clinical characteristics, biomarker profiles, and 90-day mortality.

**Results:**

Among 1392 LEDA and 1886 BASEL V patients, four reproducible subtypes were identified and labelled as ‘non-inflammatory’ (A), ‘tachycardic’ (B), ‘anaemic’ (C) and ‘hypoxemic’ (D). Subtypes transcended conventional diagnostic categories. Inflammatory and cardiovascular biomarker levels increased significantly from A to D (all *P* < .001). After adjusting for traditional risk factors, assignment remained independently associated with the 3-month mortality risk, with the worst outcome for patients assigned to the ‘hypoxemic’ subtype [adjusted HR (95% CI) for subtype D: LEDA 3.87 (2.17–6.87), *P* < .0001; BASEL V 5.15 (2.71–9.76), *P* < .001]. Adding subtype membership improved the Harrell C-index for 3-month mortality beyond a linear combination of the three main class-defining variables (LEDA 0.73 to 0.77, *P* = .04; BASEL V 0.76 to 0.79, *P* = .03).

**Conclusions:**

A model-based clustering approach identified four reproducible acute dyspnoea subtypes with distinct clinical, biomarker, and prognostic profiles. This framework may improve early risk stratification and personalized ED management beyond nosologically classified diagnoses.

## Graphical Abstract

**Graphical Abstract:**
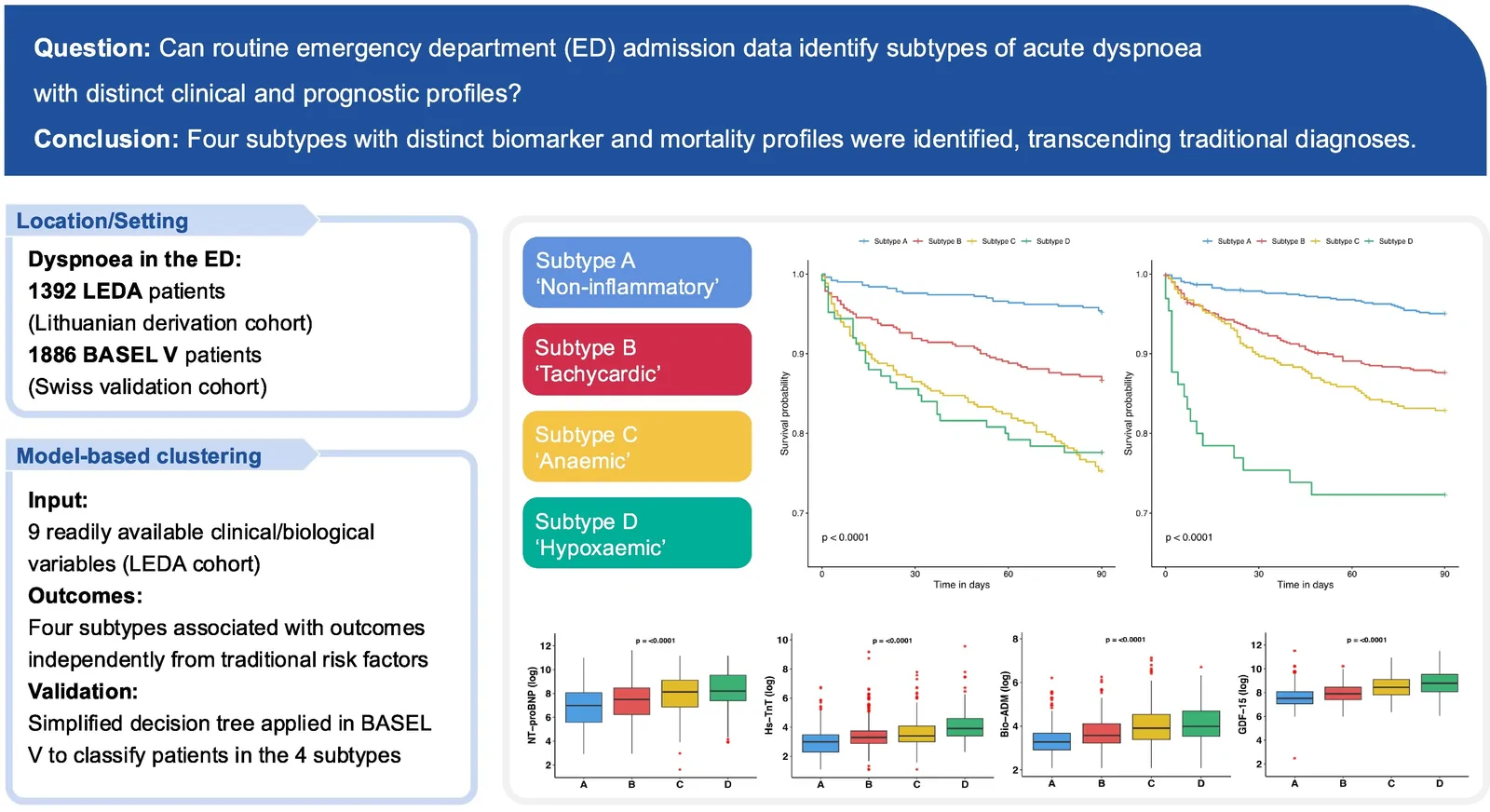
Data-driven ED subtypes of acute dyspnoea predict outcomes.

## Introduction

Acute dyspnoea is one of the most common presenting symptoms in the emergency department (ED) and a frequent cause of hospitalization.^1–3^ Emergency physicians must quickly triage, diagnose, and prognosticate, differentiating conditions ranging from acute heart failure (AHF) to cancer and assessing patient short-term risk. Inappropriate management can lead to delayed diagnosis and treatment initiation, increasing the risk of adverse clinical outcomes.^1,2,4^ This is particularly relevant in patients with acute dyspnoea, which represents a heterogeneous clinical presentation associated with substantial morbidity and mortality.^1–3,5^

Existing clinical tools for assessing dyspnoea severity, such as the New York Heart Association classification, are largely subjective and were developed for chronic or stable settings. Their reproducibility in the emergency department is limited, and they may fail to capture the severity of physiological and biochemical derangements that underlie acute dyspnoea presentations. Consequently, these scales provide only modest prognostic information and are of limited utility for early risk stratification at the time of ED admission. Given the broad differential diagnosis of acute dyspnoea, a framework that covers both AHF and non-AHF presentations are needed, as symptom severity alone does not reliably separate cardiac from non-cardiac causes.

Acute dyspnoea represents a highly diverse population unified by a common clinical symptom. Given the heterogeneity of acute dyspnoea presentations, a data-driven approach to patient phenotyping may improve risk stratification and clinical decision-making. Clinical information at presentation in the emergency department is often insufficient to reliably establish the underlying diagnosis or predict clinical trajectory, as diagnostic uncertainty is common during the initial evaluation. Traditional diagnostic frameworks may therefore incompletely capture the biological and prognostic heterogeneity of acute dyspnoea. Unsupervised learning approaches, such as latent class analysis (LCA), offer the potential to identify distinct subtypes (or clusters) within a certain patient population.^6,7^ Using readily available clinical and biochemical parameters, LCA could uncover clusters with differing biological patterns reflecting individual conditions with various prognosis.

In this study, we applied a model-based clustering LCA approach to two independent prospective cohorts of patients presenting with acute dyspnoea. Our objective was to identify clinically and biologically distinct subtypes using readily available variables and to evaluate their prognostic value independently from dyspnoea aetiology.

## Methods

A secondary analysis of two independent acute dyspnoea cohorts, LEDA and BASEL V, was performed. The analysis was first performed in the LEDA (derivation cohort) and then validated in the BASEL V (validation cohort).

### Study populations

The derivation cohort was derived from LEDA (Lithuanian Echocardiography Study of Dyspnoea in Acute Settings, *N* = 1455, NCT03048032), a prospective observational multicentre study, performed in two Lithuanian university centres in collaboration with the international GREAT network (Global Research on Acute Conditions Team, https://www.greatnetwork.org/site/). The inclusion and exclusion criteria, diagnosis adjudication, and follow-up processes have been reported elsewhere.^8^ Briefly, the study included adult patients admitted to an ED with acute dyspnoea from March 2015 to December 2017, except patients with acute coronary syndrome (ACS) suspected within the first 4 h of admission. This exclusion criterion was devised to exclude ST elevation myocardial infarction patients. Patients, who had ACS confirmed beyond the first 4 h or had ACS as an adjudicated diagnosis were not excluded from the study. The study was approved by The Lithuanian Bioethics Committee (Nr. L-15-01). AHF was defined according to the 2021 ESC Heart Failure Guidelines^1^ as new-onset or worsening heart failure requiring urgent treatment, confirmed by compatible clinical findings and elevated natriuretic peptides.^9^ CHF was defined as previously established, stable heart failure documented in the medical record or reported by the treating physician. The validation cohort was derived from BASEL V (Basics in Acute Shortness of Breath EvaLuation Study, *N* = 2153, NCT01831115), a prospective multicentre observational study. Enrolment period, inclusion/exclusion criteria, diagnosis adjudication and follow-up have been reported previously.^5,8,10^ Adult patients with acute, non-traumatic dyspnoea presenting to the ED of two university hospitals (Basel and Zurich, Switzerland) were enrolled in the study. Enrolment in BASEL V ran from April 2006 to August 2015. While enrolment was independent of renal function, patients with end-stage renal disease on chronic dialysis were excluded. The study was approved by the local ethical committees (latest site approval *KEK-ZH-Nr. 2011-0315*). Both studies were carried out according to the principles of the Declaration of Helsinki, and written informed consent was obtained from all participants.^11,12^

### Data collection and candidate variables for sub-phenotyping

Clinical and biological data were recorded at inclusion in both cohorts. Circulating inflammatory, renal, and CV biomarkers were centrally measured at inclusion in the LEDA and BASEL V studies. Details of the biomarkers and the assays were previously published and were summarized in the Appendix (Supplementary Table S1).

In the derivation LEDA cohort, clinical and biological variables readily available upon encountering a patient in the ED were considered candidate variables for inclusion in the model-based clustering analysis (i.e. LCA). The variables with more than 25% missing values were excluded from the selection process. Available clinical and biological data readily available at admission to the emergency department were selected as candidate variables based on the prior published literature.^13–15^ The final selection of variables included in the LCA models (nine variables) was made by consensus among three AHF experts: J.Č., S.S., and A.M. (Supplementary Table S2). To minimize subjective bias, variable inclusion was guided by both expert consensus and literature evidence. The final class-defining variables list consisted of systolic blood pressure (SBP), heart rate (HR), peripheral blood oxygen saturation (SpO_2_), sodium, potassium, creatinine, C-reactive protein (CRP), haemoglobin, and serum glucose concentration. Creatinine was used instead of eGFR, as eGFR equations assume steady-state renal function and may be inaccurate in acutely ill patients, whereas creatinine represents a direct measurement available at presentation. Only patients with at least five class-defining variables over nine were included in the LCA (*N* = 1392). The missing data of the nine class-defining variables and availability of the class-defining variables in the derivation cohort are presented in the Appendix (Supplementary Tables S3 and S4). Thereafter, a simplified classifier (i.e. decision trees) was developed in the derivation LEDA cohort for subtype assignment in external cohorts and potential clinical use at the bedside (Supplementary Figure S1).^16^ In the validation BASEL V cohort, patients were classified using the simplified classifier. A summary comparison of the overall baseline characteristics of the derivation and validation cohorts is provided in Supplementary Table S5.

The correlations between selected variables in the two cohorts are summarized in the Appendix (Supplementary Figure S2A and B). Clinical and biological/biomarker variables that were not included in the clustering analysis were compared across the identified patient subtypes in both cohorts.

### Study endpoints

*The primary endpoint* of this analysis was 3-month all-cause mortality. *Secondary endpoints* were 3-month cardiovascular (CV) mortality, 3-month non-cardiovascular (non-CV) mortality, 3-month all-cause, CV and non-CV readmission. Readmission was defined as hospitalization requiring at least an overnight stay. Outpatient worsening events were not systematically captured. The 3-month all-cause mortality was chosen as the primary outcome because it represents a clinically relevant and widely adopted time horizon in acute dyspnoea and heart failure research.^11,12^ Subgroup analyses regarding outcomes were performed in AHF and non-AHF patients.

Patients of the LEDA cohort were followed up for 1 year after the index episode. Data on mortality and unplanned all-cause readmissions coded by the 10th revision of the International Classification of Diseases (ICD-10) were provided by the Lithuanian administrative databases. Consequently, no patients were lost to follow-up. BASEL V patients were contacted by telephone or in written form by trained researchers at 3 months and one year after the index episode. In case of a possible relevant medical event, such as death or AHF rehospitalisation, further information was obtained from the hospital medical records, the general practitioner of the patient, or the national death registry. For the purposes of this analysis, 3-month follow-up data was used. In the LEDA cohort, cardiovascular and non-CV mortality were classified based on administrative databases and death certificates using ICD-10 codes. In the BASEL V cohort, cause of death was determined by trained researchers based on available clinical records and follow-up data, without formal independent adjudication using a predefined charter.

### Statistical analysis

Latent Class Analysis was performed as a secondary analysis using Latent Gold version 6.0 (Statistical Innovations, Arlington, MA) to identify distinct clusters/subtypes in the LEDA (derivation) cohort. The LCA was carried out independently of clinical outcomes. Missing data were addressed using Full-Information Maximum Likelihood (FIML) methods, as implemented in Latent GOLD software.^17^

Spearman’s rank-order correlation was used to assess the relationships between the class-defining variables, with highly correlated variables (IrI > 0.5) being excluded from the LCA. Non-normally distributed data were log-transformed prior to standardization (Z score) and inclusion in the LCA model. Multiple n-class models were developed, varying in the number of latent classes, ranging from one to eight.

The optimal number of latent classes was decided by considering the Bayesian Information Criteria (BIC) (lower values suggest model parsimony), class interpretability (clinical relevance) and class prevalence (classes with at least 5% of the sample). We prioritized the lowest BIC, followed by clinical significance and adequate class size. Once the optimal n-class model was selected, each patient was assigned to a single latent class based on the highest posterior probability, ensuring mutually exclusive class membership.

Decision trees were used to develop a simplified classification model (R package ‘rpart’) in the LEDA derivation cohort.^10,11^ This algorithm selected the best threshold of each class-defining variable previously included in the LCA model to maximize the discrimination of the LCA classes with a 10-fold cross-validation. The sensitivity and specificity (95% CI) of the simplified classification model to discriminate the LCA-derived classes were reported in the LEDA cohort. The Hand–Till multiclass AUC and the Brier score were used to assess the overall performance of the simplified classifier in distinguishing the four LCA classes in the LEDA cohort.^18^ Thereafter, the simplified classification model was used to discriminate the distinct classes in the validation BASEL V cohort based on the nine class-defining variables (complete-case approach, Supplementary Figure S3, *N* = 1886). Subtype characteristics were assessed and their association with outcomes was analysed in the BASEL V cohort to externally validate the classes (i.e. subtypes) generated by the simplified model.

The individual variables used in the LCA to determine the subtypes were also examined to select the best linear combination of three of them that maximized the C-index. The tertiles of these variables were then incorporated into Cox proportional hazards models to evaluate whether adding the subtypes as a variable further improved the Harrell’s C-index. For internal validation, a bootstrap resampling (2000 samples) was used.

Continuous variables are presented as medians and interquartile ranges [IQR], and categorical variables are presented as counts (percentages). Comparisons of baseline characteristics (demographic data, chronic diseases, treatment) and circulating CV, renal, and inflammatory biomarker levels between the classes were performed. The study groups were compared using the Kruskal-Wallis test for continuous variables and the χ^2^ test for categorical variables.

The impact of subtype allocation on 3-month mortality and readmission was investigated, and survival curves were generated using Kaplan–Meier analyses. Furthermore, both unadjusted and adjusted Cox proportional hazards regression analyses were performed to compare outcomes between the classes. Class allocation and variables reflecting disease severity were included in the Cox regression models. Two-sided tests were applied, with a *P*-value of ≤0.05 considered statistically significant. The analyses were performed using Latent GOLD software (v 6.0, Statistical Innovations, Arlington, MA) for LCA and SPSS 28.0 statistical software (IBM Corp., Armonk, NY, USA) and the R statistical software on R Studio (https://www.r-project.org/).

## Results

### Derivation cohort

#### Demographic and clinical data

Derivation cohort included 1392 patients (89% with at least five class-defining variables) with a median age of 71 [62–79] years, 601 (43.2%) of these patients were women. The cohort comprised of 739 (53%) AHF patients. Baseline characteristics are described in *Table 1*. 3-month all-cause mortality of the overall cohort was 13.9% (194 patients).

**Table 1:** Baseline characteristics and outcomes across the four subtypes in the derivation LEDA cohort

	Subtype A (non-inflammatory) (*n* = 499)	Subtype B (tachycardic) (*n* = 420)	Subtype C (anaemic) (*n* = 348)	Subtype D (hypoxemic) (*n* = 125)	*P* value
Age, years	69.0 [60.0, 78.0]	69.0 [61.0, 78.0]	74.0 [65.8, 81.0]	71.0 [60.0, 80.0]	<.0001
Women, *n* (%)	248 (49.7)	171 (40.7)	137 (39.4)	45 (36.0)	.0024
**Comorbidities, *n* (%)**					
History of chronic heart failure	290 (58.1)	261 (62.1)	237 (68.1)	69 (55.2)	.01
Hypertension	407 (81.6)	338 (80.5)	261 (75.0)	89 (71.2)	.02
Diabetes mellitus	75 (15.0)	134 (31.9)	65 (18.7)	44 (35.2)	<.0001
CAD	169 (33.9)	145 (34.5)	131 (37.6)	40 (32.0)	.599
Prior MI	89 (17.8)	92 (21.9)	80 (23.0)	21 (16.8)	.168
Afib/flutter	225 (45.1)	173 (41.2)	166 (47.7)	42 (33.6)	.031
COPD	45 (9.0)	76 (18.1)	37 (10.6)	24 (19.2)	.0001
Chronic renal disease	49 (9.8)	58 (13.8)	103 (29.6)	38 (30.4)	<.0001
Active/recent cancer	51 (10.2)	48 (11.4)	77 (22.1)	21 (16.8)	<.0001
Anaemia	82 (16.4)	46 (11.0)	171 (49.1)	31 (24.8)	<.0001
**Medications at admission, *n* (%)**					
ACEi/ARBs	238 (47.7)	206 (49.2)	149 (42.8)	43 (34.4)	.015
Beta blockers	266 (53.3)	212 (50.6)	167 (48.0)	53 (42.4)	.129
Diuretics^a^	196 (39.3)	187 (44.6)	164 (47.1)	53 (42.4)	.127
Aldosterone antagonists	80 (16.0)	65 (15.5)	68 (19.5)	19 (15.2)	.422
Inhaled steroids	15 (3.0)	38 (9.1)	15 (4.3)	6 (4.8)	.0005
Beta2 mimetics	25 (5.0)	51 (12.2)	22 (6.3)	10 (8.0)	.0005
Antidiabetics	47 (9.4)	74 (17.7)	35 (10.1)	22 (17.6)	.0003
**Variables used in latent class analysis, median [IQR]**					
Systolic blood pressure, mmHg	140 [128, 160]	147 [132, 164]	129 [111, 145]	131 [115, 153]	<.0001
Heart rate, bpm	80 [70, 100]	98 [80, 115]	83 [72, 97]	99 [75, 120]	<.0001
SpO2, %	95 [94, 97]	90 [87, 93]	94 [91, 96]	80 [75, 89]	<.0001
Sodium, mmol/L	141 [139, 142]	138 [136, 140]	136 [132, 140]	137 [133, 140]	<.0001
Potassium, mmol/L	4.3 [4.0, 4.5]	4.2 [3.9, 4.5]	4.4 [4.0, 4.8]	4.6 [4.1, 5.1]	<.0001
Creatinine, mcmol/L	83 [72, 100]	94 [78, 119]	115 [83, 157]	125 [82, 212]	<.0001
CRP, mg/L	4.0 [1.8, 8.4]	17.7 [6.0, 39.1]	26.0 [6.3, 61.9]	32.8 [7.6, 81.2]	<.0001
Haemoglobin, g/L	13.5 [12.7, 14.4]	13.8 [12.7, 14.9]	11.2 [9.7, 12.6]	11.5 [10.4, 14.1]	<.0001
Blood glycaemia, mmol/L	5.7 [5.2, 6.4]	7.4 [6.0, 9.6]	6.2 [5.6, 7.0]	7.4 [6.2, 12.2]	<.0001
**Other clinical parameters at admission, median [IQR]**					
BMI, kg/m^2^	29.6 [25.9, 33.9]	30.5 [25.5, 36.3]	29.1 [25.0, 33.2]	29.6 [25.4, 37.7]	.128
Borg scale of dyspnoea severity	6.0 [5.0, 8.0]	7.0 [5.0, 8.0]	7.0 [5.0, 8.0]	7.0 [5.0, 8.2]	<.0001
Duration of dyspnoea, days	7.0 [2.0, 21.0]	4.0 [1.0, 14.0]	7.0 [2.0, 14.0]	4.0 [1.0, 7.0]	.0001
Respiratory rate	17.0 [16.0, 20.0]	20.0 [18.0, 22.0]	20.0 [16.0, 22.0]	21.5 [18.0, 26.0]	<.0001
**Biomarkers, median [IQR]**					
GDF-15, ng/L	1837.5 [1169.2, 3262.5]	2692.0 [1650.0, 4729.0]	4670.0 [2462.5, 8995.0]	6576.0 [3238.0, 13860.0]	<.0001
NT-proBNP, ng/L	1071.5 [264.2, 3190.5]	1806.0 [507.0, 4725.0]	3409.0 [951.0, 9137.5]	3649.0 [1626.0, 13804.0]	<.0001
Hs-TnT, ng/L	20.0 [10.0, 32.4]	27.1 [17.8, 42.5]	30.0 [20.0, 60.0]	50.0 [30.0, 100.0]	<.0001
penKid, pmol/l	70.0 [53.4, 89.7]	68.5 [49.6, 96.0]	98.2 [65.4, 148.0]	98.7 [65.1, 165.4]	<.0001
BioADM, ng/L	26.6 [18.4, 39.7]	35.9 [25.2, 61.2]	49.8 [29.8, 93.6]	54.1 [34.3, 109.4]	<.0001
DPP3, ng/mL	104.2 [75.9, 129.7]	101.0 [76.8, 144.6]	111.6 [77.8, 165.9]	115.4 [85.7, 174.9]	.009
**Aetiology**					
AHF, *n* (%)	265 (53.1)	218 (51.9)	203 (58.3)	53 (42.4)	.02
**Outcomes**					
Hospitalized, *n* (%)	226 (45.3)	288 (68.6)	239 (68.7)	97 (77.6)	<.0001
LOS, median [IQR]^b^	8.0 [5.0, 12.0]	9.0 [7.0, 12.0]	9.0 [6.0, 14.0]	11.0 [8.0, 17.0]	<.0001
In-hospital mortality, *n* (%)	9 (1.8)	20 (4.8)	31 (8.9)	18 (14.4)	<.0001
Death at 3 months, *n* (%)	24 (4.8)	56 (13.3)	86 (24.7)	28 (22.4)	<.0001
Cardiovascular death at 3 months, *n* (%)	18 (3.6)	31 (7.4)	43 (12.4)	8 (6.4)	<.0001
Non-cardiovascular death at 3 months, *n* (%)	6 (1.2)	25 (6.0)	43 (12.4)	20 (16.0)	<.0001

ACEi/ARBs, angiotensin-converting-enzyme inhibitor/angiotensin II receptor blockers; Afib, atrial fibrillation; AHF, acute heart failure; bioADM, bioactive adrenomedullin; BMI, body mass index; bpm, beats per minutes; CAD, coronary artery disease; COPD, chronic obstructive pulmonary disease; CRP, C-reactive protein; DPP3, circulating dipeptidyl peptidase; GDF-15, growth-differentiation factor-15; HGB, haemoglobin; Hs-TnT, high-sensitive troponin-T; IQR, interquartile range; LOS, length of hospital stay; MI, myocardial infarction; NT-pro-BNP, N-terminal pro-B-type natriuretic peptide; penKid, Proenkephalin A 119–159; SpO2, peripheral oxygen saturation.

^a^Any diuretic.

^b^Only hospitalized patients.

#### Identification of patient subtypes

A four-class LCA model of patients presenting to the ED with dyspnoea was selected as the optimal fit clustering solution. A summary of the model fits for one to eight classes for the LEDA cohort is provided in the Appendix (Supplementary Figure S4). Model selection favoured the four-class solution, which had the lowest Bayesian Information Criterion; the corresponding entropy was 0.63 and the median [IQR] posterior class-membership probability was 0.84 [0.75–0.96], indicating adequate class separation and assignment certainty (Supplementary Table S7). A chronological 60%/40% split-sample sensitivity analysis of the LEDA cohort (internal derivation set, *N* = 835; internal validation set, *N* = 557) is presented in Supplementary Tables S8–S11 and Supplementary Figures S11 and S12.

Subtypes were labelled according to their predominant distinguishing clinical characteristic. Hence, upon presentation to the ED, 499 (36%) patients were assigned to subtype A (‘non-inflammatory’), 420 (30%) to subtype B (‘tachycardic’), 348 (25%) to subtype C (‘anaemic’) and 125 (9%) to subtype D (‘hypoxemic’).

#### Clinical and biomarker profiles of the patient subtypes

**Figure 1:**
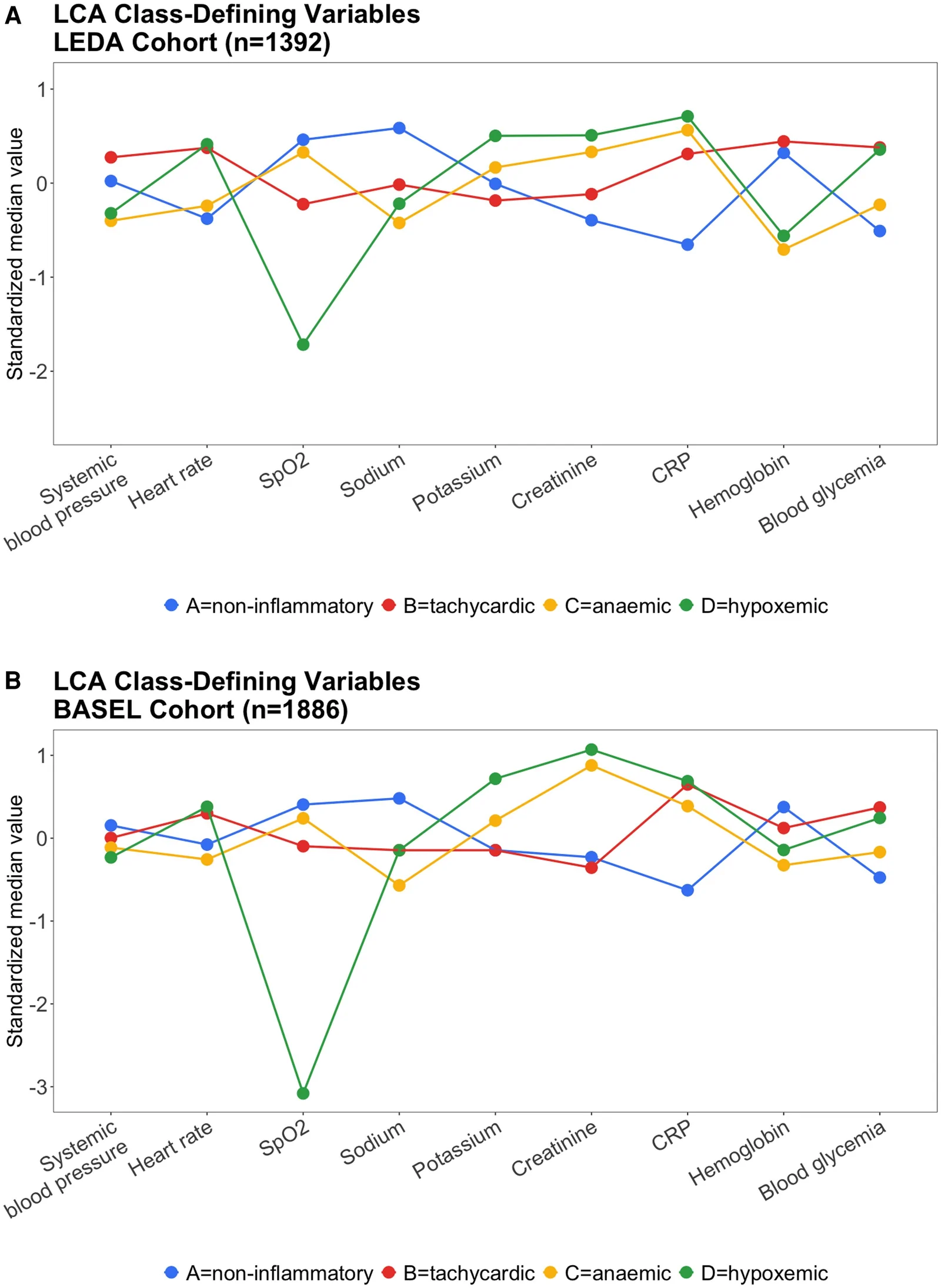
Comparison of class-defining variables using latent class analysis at inclusion in the derivation LEDA cohort (*A*) and using the simplified classifier at inclusion in the validation BASEL V cohort (*B*). Description: continuous variables were plotted after natural log transformation. Every normalized variable was standardized such that all means are scaled to 0 and SDs to 1. Group means of standardized values are shown by subtypes. A value of +1 for the standardized variable (*y*-axis) indicates that the mean value for a given subtype was one SD higher than the mean value in a whole cohort. Subtype labels: A—‘non-inflammatory’, B—‘tachycardic’; C—‘anaemic’ and D—‘hypoxemic’. CRP, C reactive protein; SD, standard deviation; SpO2, peripheral oxygen saturation

The comparison of mean standardized values of class-defining variables at inclusion across the four subtypes of the LEDA cohort is displayed in *Figure 1A*. *The class-defining variables differed significantly across subtypes in both cohorts* (*Tables 1* and *2; Figure 1*). A radar (spider) plot further illustrates the overlapping but distinct multidimensional profiles of the four subtypes (Supplementary Figure S10). Of the variables used in LCA, the non-inflammatory subtype was characterized by the lowest CRP, the lowest creatinine, lowest prevalence of diabetes mellitus and the highest oxygen saturation. Patients in the tachycardic subtype had a high heart rate and high blood pressure. The anaemic subtype was older, had the highest prevalence of malignancy and presented the lowest levels of haemoglobin, sodium, and blood pressure. The hypoxemic subtype had the lowest oxygen saturation, showed the greatest male proportion along with the highest creatinine, potassium, CRP and blood glycaemia.

**Table 2:** Baseline characteristics and outcomes across subtypes in the validation cohort (BASEL V)

	Cluster A (*n* = 752)	Cluster B (*n* = 701)	Cluster C (*n* = 368)	Cluster D (*n* = 65)	*P*-value
Age (years)	73 [59, 82]	74 [62, 82]	79 [70, 84]	77 [68.5, 83]	< .001
Women, *n* (%),	341 (45.3%)	319 (45.5%)	151 (41.0%)	22 (33.8%)	.157
Chronic heart failure, *n* (%)	218 (29.0%)	195 (27.9%)	175 (47.6%)	29 (44.6%)	< .001
Diabetes mellitus, *n* (%)	105 (14.0%)	216 (30.8%)	100 (27.2%)	25 (38.5%)	< .001
Hypertension, *n* (%)	492 (65.6%)	467 (66.7%)	291 (79.1%)	46 (70.8%)	< .001
Prior MI, *n* (%)	143 (19.0%)	107 (15.3%)	98 (26.6%)	19 (29.2%)	< .001
Afib/flutter, *n* (%)	183 (24.3%)	179 (25.5%)	128 (34.8%)	29 (44.6%)	< .001
CAD, *n* (%)	247 (32.9%)	204 (29.1%)	170 (46.2%)	26 (40.0%)	< .001
Chronic renal disease, *n* (%)	164 (21.8%)	134 (19.1%)	218 (59.2%)	35 (53.8%)	< .001
Active/recent cancer, *n* (%)	26 (3.9%)	24 (4.0%)	25 (7.5%)	1 (1.7%)	.03
**Variables used in latent class analysis, median [IQR]**					
Systolic Blood Pressure (mmHg)	140 [124, 156]	136 [120, 154]	133 [114, 153]	130 [105, 148]	< .001
Heart Rate (bpm)	88 [73, 100]	97 [82, 112]	84 [71, 100]	99 [81, 110]	< .001
SpO2 (%)	97 [95, 99]	94 [90, 97]	96 [94, 99]	78 [73, 85]	< .001
Sodium (mmol/L)	140 [138, 142]	137 [135, 140]	136 [133, 139]	137 [135, 141]	< .001
Potassium (mmol/L)	4.0 [3.7, 4.3]	4.0 [3.7, 4.4]	4.2 [3.9, 4.6]	4.5 [4.2, 4.9]	< .001
Creatinine (µmol/L)	84 [68, 103]	79 [64, 97]	144 [110, 196]	158 [102, 213]	< .001
CRP (mg/L)	4.6 [2.4, 10.4]	39.0 [9.3, 95.2]	24.0 [9.6, 60.8]	39.0 [16.1, 138.9]	< .001
Hemoglobin (g/L)	138.0 [124.0, 149.0]	132.0 [120.0, 145.0]	122.0 [109.0, 138.0]	126.0 [114.0, 147.0]	< .001
Blood glycaemia (mmol/L)	6.1 [5.4, 7.0]	7.8 [6.1, 10.1]	6.6 [5.8, 7.9]	7.5 [6.3, 9.9]	< .001
**Biomarkers**					
GDF-15 (ng/L)	2123 [1203, 3925]	2855 [1705, 4883]	5659 [3046, 10622]	6706 [3833, 13494]	< .001
NT-proBNP (ng/L)	842 [117, 3204]	1085 [276, 3979]	4477 [1104, 10503]	5763 [1756, 13579]	< .001
hs-TnT (ng/L)	17.0 [8.0, 37.0]	22.0 [12.0, 41.5]	41.0 [24.0, 70.0]	42.0 [28.0, 88.0]	< .001
BioADM (ng/L)	22.9 [15.6, 34.5]	31.7 [21.5, 45.7]	42.4 [26.9, 70.8]	47.8 [34.1, 93.1]	< .001
proENK (pmol/L)	74.6 [60.2, 128.3]	62.4 [33.3, 124.5]	346.9 [121.9, 431.8]	217.0 [76.4, 223.1]	.121
IL-6 (pg/mL)	5.4 [2.6, 10.4]	19.2 [7.8, 56.5]	16.8 [8.5, 38.5]	33.1 [12.9, 212.0]	< .001
**Outcomes**					
In-hospital mortality, *n* (%)	12 (1.6)	43 (6.1)	27 (7.3)	16 (24.6)	< .001
LOS, median [IQR]	5.0 [0.0, 12.0]	9.0 [4.0, 15.0]	12.0 [7.0, 19.0]	14.0 [6.5, 22.5]	< .001
90-day mortality, *n* (%)	37 (4.9)	86 (12.3)	63 (17.1)	18 (27.7)	< .001
Cardiovascular 90-day mortality, *n* (%)	23 (3.1)	37 (5.3)	35 (9.5)	8 (12.3)	< .001
Non-cardiovascular 90-day mortality, *n* (%)	14 (1.9)	49 (7.0)	28 (7.6)	10 (15.4)	< .001

Data are presented as medians [IQR] or counts (%).

Afib, atrial fibrillation; BioADM, bioactive adrenomedullin; bpm, beats per minute; CAD, coronary artery disease; CRP, C-reactive protein; GDF-15, growth differentiation factor 15; hs-TnT, high-sensitivity troponin T; IL-6, interleukin 6; IQR, interquartile range; LOS, length of hospital stay; MI, myocardial infarction; NT-proBNP, N-terminal pro–B-type natriuretic peptide; proENK, proenkephalin; SpO_2_, peripheral capillary oxygen saturation.

**Figure 2:**
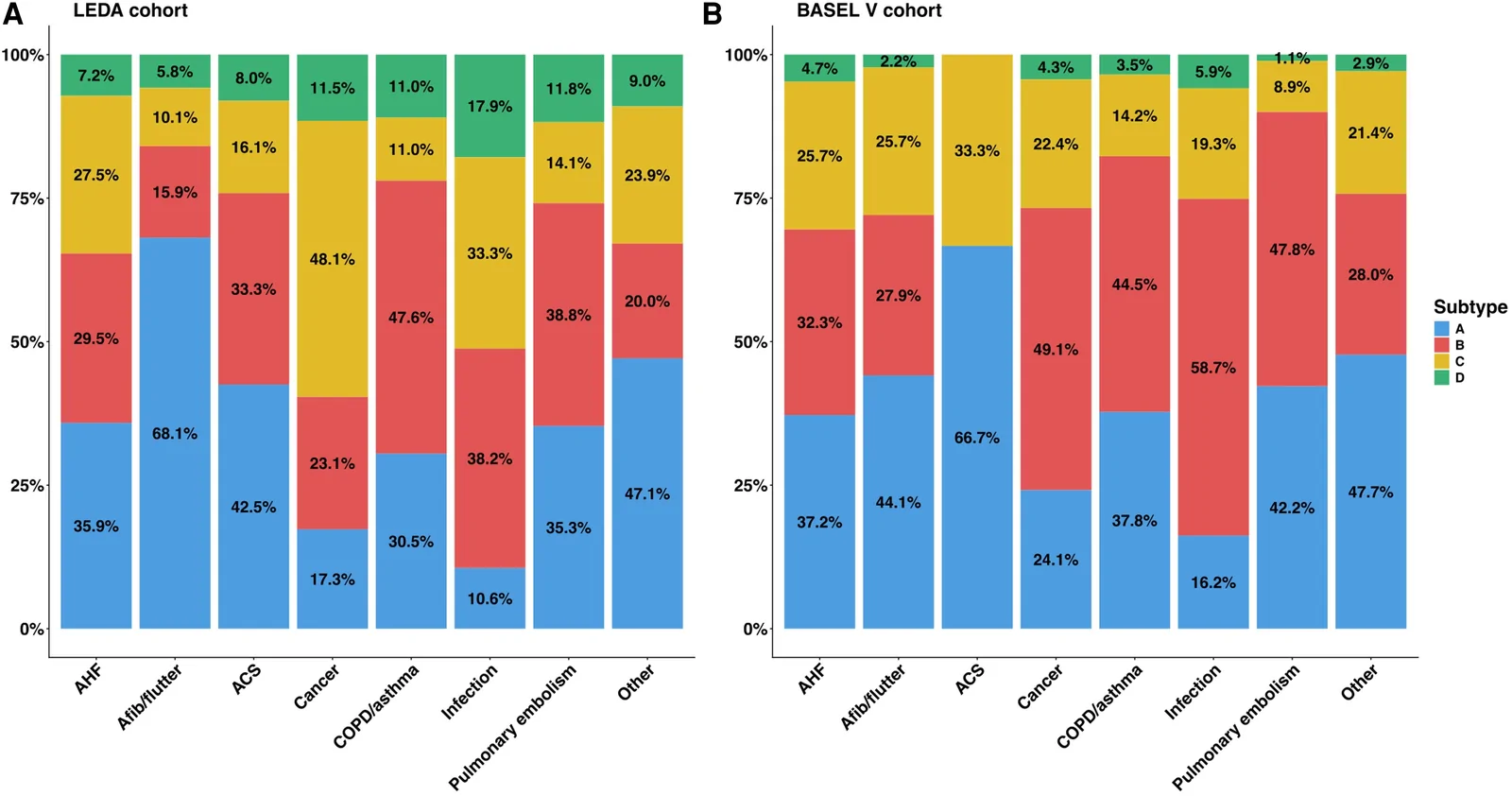
Distribution of the four subtypes in groups of adjudicated diagnoses in the derivation LEDA cohort (*A*) and the validation BASEL V cohort (*B*). Subtype labels: A—‘non-inflammatory’, blue; B—‘tachycardic’, red; C—‘anaemic’, yellow and D—‘hypoxemic’, green. ACS, acute coronary syndrome; Afib, atrial fibrillation; AHF, acute heart failure; COPD, chronic obstructive pulmonary disease

Baseline, clinical/biological characteristics and outcomes across subtypes in the LEDA cohort are summarized in *Table 1*. The distribution of the four subtypes among the groups of adjudicated diagnoses on admission is summarized in *Figure 2A*.

**Figure 3:**
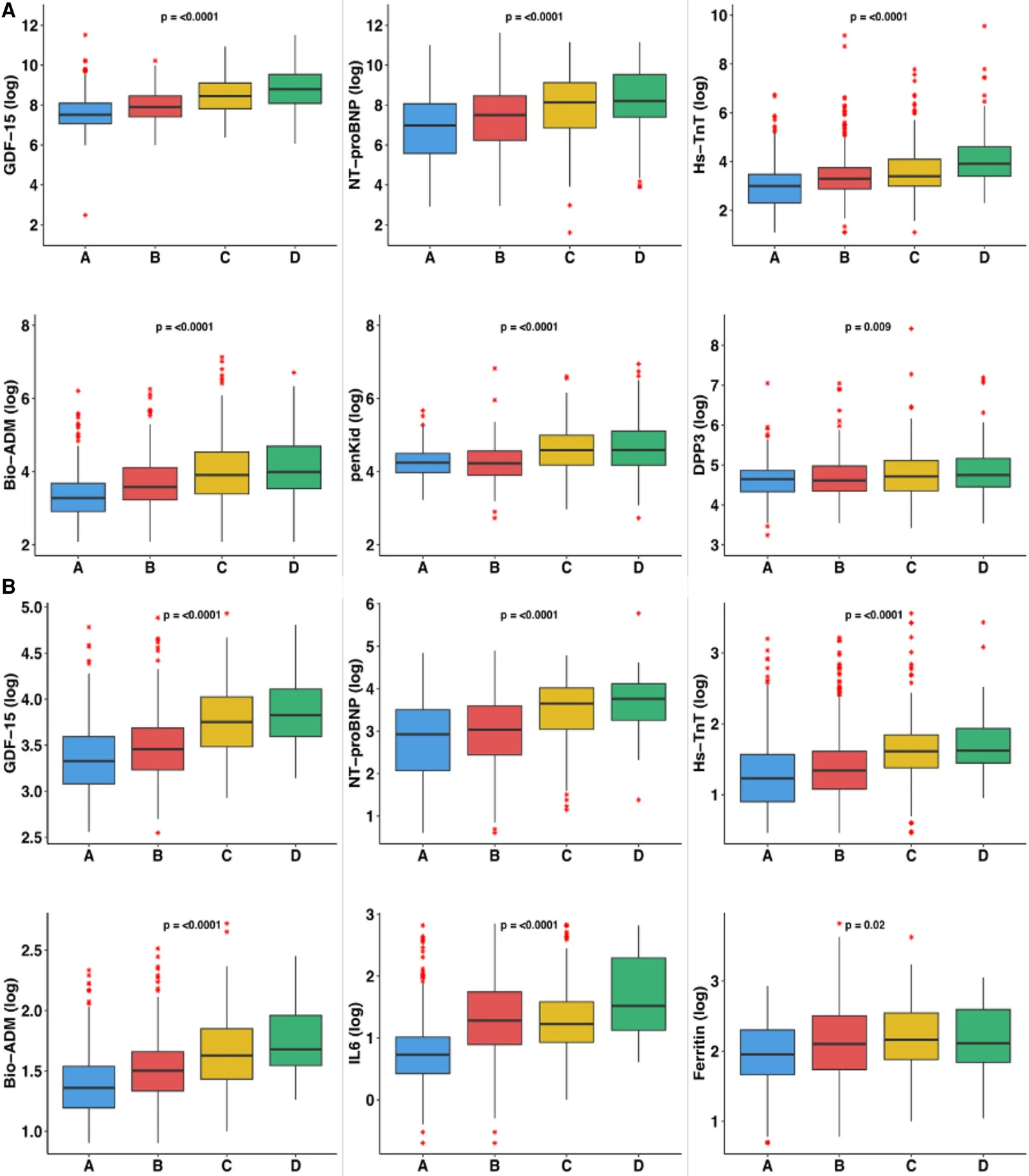
Differences in biomarker concentrations across the four subtypes in the derivation LEDA cohort (*A*) and the validation BASEL V cohort (*B*). bioADM, bioactive adrenomedullin; DPP3, circulating dipeptidyl peptidase; GDF-15, growth-differentiation factor-15; Hs-TnT, high-sensitive troponin-T; NT-proBNP, *N*-terminal pro-B-type natriuretic peptide; penKid, Proenkephalin A 119–159. Subtype labels: A—‘non-inflammatory’, B—‘tachycardic’; C—‘anaemic’ and D—‘hypoxemic’

A consistent gradient of biomarker levels was observed from the lowest levels in the non-inflammatory subtype to the highest in the hypoxemic subtype (*Table 1*, *Figure 3*). Remarkably, it was true for CV, renal as well as inflammatory markers: N-terminal pro–B-type natriuretic peptide (NT-proBNP), high-sensitivity Troponin T (hs-TnT), growth differentiation factor-15 (GDF-15), bioactive adrenomedullin (bioADM), Proenkephalin A 119–159 (penKid) and circulating dipeptidyl peptidase (DPP3).

#### Association between dyspnoea subtypes and outcomes

The four identified patient subtypes significantly differed in terms of prognosis. The outcomes across the different subtypes are summarized in *Table 1*. Patients in the non-inflammatory subtype were the least likely to be hospitalized [226 patients (45.3%)], while the rate of hospitalization from the ED was the highest in the hypoxemic subtype [97 patients (77.6%), *P* < .0001]. The length of stay (LOS) for hospitalized patients differed significantly across the four subtypes, with the longest duration in the hypoxemic subtype. In-hospital mortality was the highest in the hypoxemic subtype.

**Figure 4:**
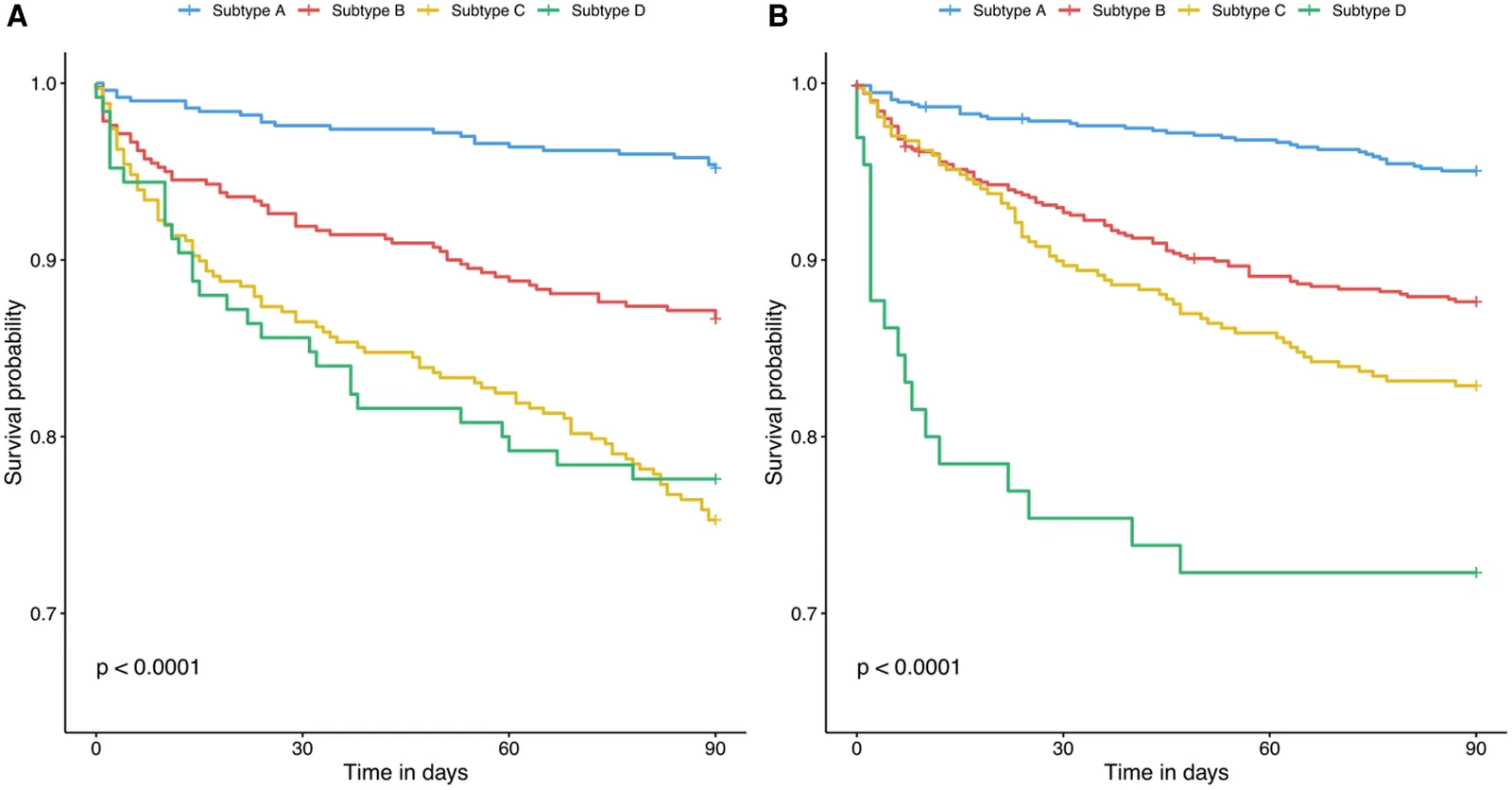
3-month all-cause mortality in acute dyspnoea cohorts based on the four subtypes in (*A*) the derivation LEDA cohort and (*B*) the validation BASEL V cohort. Subtype labels: A—‘non-inflammatory’, B—‘tachycardic’; C—‘anaemic’ and D—‘hypoxemic’

All-cause 3-month mortality was the lowest in the non-inflammatory subtype [24 patients (4.8%)], while it was the highest in subtypes C and D [86 (24.7%) and 28 (22.4%), respectively, *P* < .0001, *Table 1*, *Figure 4A*]. Regarding the cause of death, patients in the non-inflammatory subtype were more likely to die of CV causes, the tachycardic and anaemic subtypes had similar rates of CV and non-CV mortality. In contrast, patients in the hypoxemic subtype were significantly more likely to die of non-CV causes (*Table 1*, Supplementary Figure S5A and B). Notably, the dependence of all-cause 3-month mortality on the assigned subtype remained significant after adjusting for traditional risk factors such as age, sex, history of chronic heart failure, diabetes, cancer, COPD, chronic kidney disease, hospitalization from the ED on admission and adjudicated diagnosis (*Table 3*).

**Table 3:** Unadjusted and adjusted Cox regression for 3-months all-cause mortality in the derivation LEDA cohort

A. Unadjusted analysis	HR (95% CI)	*P* value
Subtype A (non-inflammatory)	1 (1–1)	Reference
Subtype B (tachycardic)	2.93 (1.81–4.72)	<.0001
Subtype C (anaemic)	5.72 (3.64–9)	<.0001
Subtype D (hypoxemic)	5.25 (3.04–9.05)	<.0001

B. Adjusted analysis	HR (95% CI)	*P* value
Subtype A (non-inflammatory)	1 (1–1)	Reference
Subtype B (tachycardic)	2.48 (1.51–4.05)	.0003
Subtype C (anaemic)	3.57 (2.22–5.76)	<.0001
Subtype D (hypoxemic)	3.87 (2.17–6.87)	<.0001
Age per 10 years	1.24 (1.09–1.41)	.001
Female sex	0.93 (0.68–1.26)	.632
History of CHF	1.15 (0.81–1.63)	.443
History of diabetes	0.74 (0.5–1.09)	.123
History of cancer	2.38 (1.67–3.38)	<.0001
History of COPD	0.98 (0.63–1.52)	.917
History of chronic kidney disease	1 (0.69–1.44)	.991
Other diagnosis at admission	1 (1–1)	Reference
AHF at admission	0.92 (0.51–1.66)	.790
Infection at admission	1.79 (0.93–3.43)	.08
COPD or asthma at admission	1.53 (0.68–3.47)	.307
Cancer at admission	3.07 (1.53–6.14)	.0016
Pulmonary embolism at admission	1.02 (0.44–2.33)	.967
Afib/flutter at admission	0.18 (0.02–1.38)	.0989
ACS at admission	1.26 (0.57–2.79)	.574
Hospitalized at admission	1.37 (0.98–1.9)	.064

ACS, acute coronary syndrome; Afib, atrial fibrillation; AHF, acute heart failure; CHF, chronic heart failure; COPD, chronic obstructive pulmonary disease.

All-cause and non-CV 3-month readmission rates were the highest in subtype C. There were no significant differences between subtypes in 3-month CV readmissions (Supplementary Figure S6).

#### Subgroup analysis

After stratification of patients into AHF and non-AHF subgroups, the differences in all-cause mortality, as well as CV and non-CV mortality, between the four clusters were still observed, as displayed in Supplementary Figures S7 and S8. The same was true for the composite endpoint of 3-month all-cause readmission or mortality (Supplementary Figure S9).

### Validation cohort

The simplified classifier reproduced the four LCA-derived subtypes with good performance in the LEDA cohort [Hand–Till multiclass AUC (95% CI) 0.87 (0.85–0.88); Brier score 0.19; the class-defining variable patterns of the four subtypes are shown in Supplementary Figure S13] and was applied to classify patients in the BASEL V validation cohort.

#### Demographic and clinical data

A total of 1886 patients from the validation cohort had data available for LCA and were eligible for this analysis (Supplementary Figure S3). Patients were older with a median age of 75 [63–82] years, similar to the derivation cohort 833 (44%) were women (*Table 2*). A history of chronic heart failure was present in 617 patients (33%) while the distribution of AHF was comparable to the derivation cohort [975 patients (52%) with AHF].

#### Clinical and biomarker profiles of the patient subtypes

The patient characteristics based on subtype classes are summarized in *Table 2*. Overall, the four subtypes identified in the BASEL V validation cohort exhibited clinical and biological characteristics, as well as a mortality risk (i.e. a higher mortality in the ‘hypoxemic’ the hypoxemic subtype), similar to the corresponding LCA-derived subtypes in the LEDA cohort (*Figure 1B*). The distribution of the four subtypes in the groups of adjudicated diagnosis on admission is summarized in *Figure 2B*.

The gradient of plasma concentrations of circulating biomarkers, observed in the derivation cohort, was replicated in the BASEL V validation cohort, which included NT-proBNP, hsTnT, GDF-15, bioADM, interleukin 6 (IL-6), and ferritin (*Figure 3B*). Patients in the hypoxemic subtype (‘hypoxemic’) had the highest concentration of all six measured biomarkers.

#### Association between patient subtypes and outcomes

At 3 months of follow-up, 204 (10.8%) patients had died, and no patients were lost to follow-up. All-cause 3-month mortality was lowest in the non-inflammatory subtype (37 patients, 4.9%), higher in the tachycardic subtype (86 patients, 12.3%), and highest in the anaemic and hypoxemic subtypes and (63 patients, 17.1%, and 47 patients, 27.7%, respectively; *P* < .001, *Figure 4B*). The non-inflammatory and tachycardic subtypes exhibited comparable rates of CV and non-CV mortality, whereas subtype anaemic had a higher rate of CV mortality. In contrast, patients in the hypoxemic subtype were significantly more likely to die from non-CV causes (Supplementary Figure S5C and D).

Subtype assignment demonstrated independent prognostic value for predicting all-cause 3-month mortality after adjusting for the traditional risk factors, including age, sex, history of chronic heart failure, diabetes, cancer, COPD, chronic kidney disease, hospitalization from the ED at admission, and adjudicated diagnosis (*Table 4*).

**Table 4:** Unadjusted and adjusted Cox regression for 3-months all-cause mortality in the validation BASEL V cohort

A. Unadjusted analysis	HR (95% CI)	*P* value
**Subtype A (non-inflammatory)**	1 (1–1)	Reference
**Subtype B (tachycardic)**	2.61 (1.77–3.83)	<.001
**Subtype C (anaemic)**	3.68 (2.45–5.53)	<.001
**Subtype D (hypoxemic)**	6.89 (3.93–12.11)	<.001

B. Adjusted analysis	HR (95% CI)	
**Subtype A (non-inflammatory)**	1 (1–1)	Reference
**Subtype B (tachycardic)**	2.47 (1.59–3.83)	<.001
**Subtype C (anaemic)**	2.23 (1.41–3.51)	<.001
**Subtype D (hypoxemic)**	5.15 (2.71–9.76)	<.001
Age per 10 years	1.44 (1.23–1.69)	<.001
Male sex	1.30 (0.94–1.78)	.109
History of CHF	1.43 (1.02–2.02)	.041
History of diabetes	0.54 (0.37–0.80)	.002
History of cancer	1.19 (0.77–1.82)	.434
History of COPD/Asthma	1.20 (0.81–1.77)	.377
History of chronic kidney disease	1.54 (1.07–2.20)	.019
Other diagnosis at admission	1.59 (1.02–2.47)	.04
AHF at admission	1.47 (0.93–2.32)	.099
Infection precipitating AHF	1.24 (0.85–1.81)	.272
COPD or asthma at admission	0.60 (0.35–1.01)	.055
Cancer at admission	7.66 (4.58–12.80)	<.001
Pulmonary embolism at admission	0.41 (0.10–1.69)	.217
Afib/flutter precipitating AHF	0.63 (0.34–1.17)	.142
ACS and AHF at admission	1.98 (1.11–3.54)	.021

AHF, acute heart failure; Afib, atrial fibrillation; ACS, acute coronary syndrome; CHF, chronic heart failure; CI, confidence interval; COPD, chronic obstructive pulmonary disease; HR, hazard ratio.

In a second Cox regression model including tertiles of SBP, SpO2 and CRP (The three variables selected which provided the highest C-index) and the same covariates as in *Table 3* (LEDA cohort) and *Table 4* (BASEL V cohort), the subtypes improved the Harrell’s C-index showing that they added value to models beyond that what could be obtained from a simple linear combination of the variables themselves (Supplementary Table S6).

#### Comparison of the subtypes across the derivation (LEDA) and validation (BASEL V) cohorts

To allow a direct, symmetrical comparison, the four subtypes are described in parallel below, reporting the derivation (LEDA) and validation (BASEL V) cohorts in turn for each subtype.

##### Subtype A (‘non-inflammatory’)

In the LEDA derivation cohort (*n* = 499), this subtype had the lowest CRP and creatinine, the highest oxygen saturation and the lowest prevalence of diabetes, and carried the best prognosis (3-month all-cause mortality 24 patients, 4.8%). In the BASEL V validation cohort, the corresponding subtype reproduced this low-inflammatory, well-oxygenated profile and again had the lowest mortality (37 patients, 4.9%).

##### Subtype B (‘tachycardic’)

In LEDA (*n* = 420), patients had a high heart rate and high SBP with intermediate biomarker levels and an intermediate outcome (56 patients, 13.3%). In BASEL V, the corresponding subtype reproduced this tachycardic, hypertensive profile and a comparable, intermediate mortality (86 patients, 12.3%).

##### Subtype C (‘anaemic’)

In LEDA (*n* = 348), patients were older, had the highest prevalence of malignancy and the lowest haemoglobin, sodium and blood pressure, with a high mortality (86 patients, 24.7%). In BASEL V, the corresponding subtype was likewise anaemic and older, and again showed a high mortality (63 patients, 17.1%).

##### Subtype D (‘hypoxemic’)

In LEDA (*n* = 125), this subtype had the lowest oxygen saturation and the highest creatinine, potassium, CRP and glycaemia, and the worst prognosis [28 patients, 22.4%; adjusted HR 3.87 (2.17–6.87)]. In BASEL V, the corresponding subtype again showed the most severe hypoxaemia and multi-organ derangement and the highest mortality [47 patients, 27.7%; adjusted HR 5.15 (2.71–9.76)].

Across both cohorts, a consistent gradient of inflammatory, renal and cardiovascular biomarkers was observed from subtype A to subtype D, and subtype assignment remained independently associated with 3-month mortality after adjustment for traditional risk factors, with a concordant mortality gradient (A < B < C ≈ D). This parallel behaviour supports the reproducibility of the subtypes across the derivation and validation cohorts.

## Discussion

In this study, we applied a model-based clustering approach to the prospective observational multicentre cohort of acute dyspnoea patients (LEDA trial) and identified four distinct subtypes of patient profiles, characterized by a gradient of CV, renal, and inflammatory biomarkers, as well as different prognosis. These findings were verified in the independent prospective observational cohort BASEL V. Based on the clinical and biological profiles, the subtypes were labelled as A—‘non-inflammatory’, B—‘tachycardic’, C—‘anaemic’ and D—‘hypoxemic’. After adjusting for traditional risk factors, assignment to one of four clusters remained independently associated with the 3-month mortality risk, with the best outcomes for patients in A (‘non-inflammatory’) and the worst for those in D (‘hypoxemic’) subtypes. The results of our study underscore the heterogeneity of patients admitted to the ED with acute dyspnoea above their age, comorbidities, the need for admission and the primary diagnosis causing dyspnoea. The identified subtypes reflect distinct clinical and biological profiles characterized by consistent differences in biomarker levels and physiological parameters, which were also associated with differences in prognosis across both cohorts.

Our objective was not to identify an AHF–specific dyspnoea phenotype, but rather to identify common biological subtypes among patients presenting with acute dyspnoea, irrespective of the underlying aetiology. This approach is consistent with the emerging concept that biological response patterns may transcend traditional diagnostic categories.^19^ Accordingly, the identified clusters were not expected to represent specific cardiac dyspnoea phenotypes. The AHF/non-AHF analyses were performed to assess the distribution and prognostic relevance of these subtypes across conventional diagnostic groups. Natriuretic peptides were not included among the class-defining variables because our aim was not to build a diagnostic model separating AHF from non-AHF dyspnoea, a distinction that BNP/NT-proBNP already strongly capture in routine care; their inclusion would likely have driven the clustering primarily toward this known separation rather than revealing cross-aetiology biological patterns. The subtype labels (‘non-inflammatory’, ‘tachycardic’, ‘anaemic’ and ‘hypoxemic’) are pragmatic descriptors of the predominant biological pattern within each cluster, each defined by a combination of class-defining variables rather than a single dominant feature or a definitive mechanistic diagnosis. We have clarified this rationale in the revised manuscript.

To our knowledge, this is among the first studies to apply unsupervised learning for identifying distinct clusters or subtypes in a heterogeneous group of ED patients with acute dyspnoea. This method has been applied in other populations associated with acute dyspnoea. For example, phenomapping using unsupervised clustering has been applied to identify novel subtypes of heart failure with preserved ejection fraction.^20^ Investigators of RELAX-AHF-2 (Efficacy, Safety and Tolerability of Serelaxin When Added to Standard Therapy in AHF) used LCA to define multimorbidity clusters in AHF.^21^ They used patients’ history of comorbidities as clustering parameters and found five distinct multimorbidity clusters with different clinical outcomes. Model-based clustering of patients, revealing unique patterns of comorbidities’ combinations, provided more personalized prognostication; moreover, significant interaction of patient cluster with treatment allocation was found. Similarly, in our study LCA allowed to create a new classification of patient subtypes differing in the disease and outcome severity, based on readily available admission variables. In the RELAX-AHF-2 study, the worst outcomes were observed in patients with diabetes and CKD as their main comorbidities. This was also apparent in our cohort as patients in the D subtype (‘hypoxemic’) with baseline hyperglycaemia and impaired renal function had the worst prognosis. These clusters likely reflect pathophysiological mechanisms contributing to acute dyspnoea severity. Elevated CRP, creatinine, and glucose in the hypoxemic subtype suggest impaired tissue perfusion and metabolic derangement, whereas lower inflammatory and renal markers in the non-inflammatory subtype indicate better preserved end-organ function. LCA has also been used to define two distinct subtypes (hyperinflammatory and hypoinflammatory) in acute respiratory failure patients.^7,22^ Patients in the hyperinflammatory cluster, characterized by higher plasma levels of inflammatory biomarkers, a higher prevalence of vasopressor use, lower serum bicarbonate, and a higher prevalence of sepsis, experienced significantly worse outcomes than the non-inflammatory patients. In our study, the subtype characterized by a low level of CRP was also identified (subtype A, or ‘non-inflammatory’), which exhibited the lowest levels of other CV and renal circulating markers as well as the best prognosis.

Striking differences were observed between the subtypes in our study. Although some individual features overlapped between subtypes, each subtype was defined by a distinct combination of physiological and laboratory abnormalities. For example, two subtypes with high blood glycaemia and a similar proportion of diabetes were subtypes B (‘tachycardic’) and subtypes D (‘hypoxemic’). The proportion of adjudicated underlying causes of the acute dyspnoea was also comparable between these two subtypes. However, the peripheral oxygen saturation, creatinine and CRP levels were significantly different in the two subtypes, exhibiting vastly different outcomes. Another example would be subtypes A (‘non-inflammatory’) and C (‘anaemic’), where glucose and oxygenation levels were comparable but they differed greatly in CRP and haemoglobin concentration and had significantly different prognosis even after multivariate adjustment.

Patients in both cohorts were recruited in the ED, where acute dyspnoea is one of the most common symptoms, which presents a tremendous challenge to the strained emergency physicians.^16^ Acute dyspnoea reflects a heterogeneous clinical population in emergency care, where symptoms represent the primary clinical information available at presentation before the underlying diagnosis is established. The present analysis identified reproducible patient groups based on objective clinical and laboratory parameters available at presentation, which were associated with distinct clinical profiles and outcomes across two independent cohorts. These findings could potentially be used as a new and simple tool to better classify acute dyspnoea in the ED and improve risk stratification. Nonetheless, the value of such tools needs to be confirmed in a prospective study.^22^

From a clinical perspective, the identified subtypes may support more tailored early management in the ED. Patients with the poorest prognosis showed the highest inflammatory and renal stress biomarker levels, suggesting the need for prompt recognition of possible life-threatening conditions with multi-organ compromise and prioritization for monitoring or escalation of care. While these observations are hypothesis-generating, they illustrate how simple admission parameters could help refine early triage and resource allocation in patients with acute dyspnoea. Accordingly, these subtypes could be integrated into early triage and management pathways. Importantly, these subtypes are intended to support early risk stratification rather than replace diagnostic evaluation, and treatment decisions should remain based on the underlying cause of dyspnoea. Beyond prognostic enrichment, they may also enable predictive enrichment by identifying patients who respond differently to interventions (e.g. high-flow nasal cannula or non-invasive ventilation) despite similar baseline risk.

These findings should nevertheless be interpreted with caution. The identified subtypes may, in part, reflect a gradient of illness severity rather than fully distinct biological phenotypes; in particular, the ‘hypoxemic’ subtype appears to capture patients with multi-organ dysfunction. Accordingly, the subtypes are proposed as a pragmatic aid to early risk stratification rather than as definitive disease entities, and over interpretation of the descriptive labels should be avoided.

The main strength of our analysis is the use of LCA for deriving and validating distinct patient subtypes in two independent prospective acute dyspnoea cohorts using readily available variables. However, our work has a few limitations. First, the analysed patients were enrolled in European EDs. Therefore, the results may lack applicability in other regions, where patient demographics, disease prevalence, and healthcare resource availability differ substantially. Validation in settings with different triage systems and access to laboratory testing will be needed to confirm external generalizability. Nonetheless, because the subtypes are derived from routinely collected, aetiology-independent physiological and laboratory variables, the approach is, in principle, transferable to other healthcare systems, including resource-limited settings, once the subtype distribution and prognostic associations are re-examined locally. Second, due to the observational nature of the cohorts, there is some missing data. Nonetheless, the range of missingness (%) by class-defining variable was between 0% and 25% and was handled using appropriate statistical methods.^18,23–25^ Third, both cohorts included patients before the COVID-19 pandemic, which has since impacted the workload in EDs and greatly increased the number of patients presenting with acute dyspnoea. However, our study showed that even before the pandemic, patients with increased inflammatory biomarkers and ultimately diagnosed with infection had worse outcomes. As the subtypes capture aetiology-independent biological responses rather than specific diagnoses, they are likely to remain applicable to the evolving case-mix of acute dyspnoea encountered after the pandemic, although confirmation in contemporary cohorts is warranted. Fourth, the analysis of a potential heterogeneity of treatment effect across the four subtypes was not possible as no randomized controlled trials of patients with acute dyspnoea were included in the analysis. Fifth, both cohorts required written informed consent. Therefore, small selection bias towards the enrolment of patients eligible to provide it was unavoidable. Sixth, while the identified subtypes provided prognostic stratification, treatment decisions remain guided by the underlying diagnosis and current guidelines. Whether subtype-based stratification could inform therapeutic strategies requires further research. Seventh, chest X-ray, ECG, echocardiography, and lung ultrasound contributed to diagnostic adjudication but were not uniformly available at presentation in the LEDA derivation cohort. The model was therefore restricted to clinical and laboratory variables consistently collected at emergency department presentation. Eight, glucose measurements were obtained at emergency department presentation and were therefore non-fasting, which may have introduced variability related to acute stress and recent food intake. Ninth, patients with suspected ACS in the LEDA cohort and those receiving chronic dialysis in the BASEL V cohort were excluded according to the original study protocols, which may limit generalisability to these populations. Tenth, outpatient worsening events were not systematically collected and could therefore not be included in the analysis. Finally, while 3-month outcomes are well established as an indicator of early post-discharge risk, longer follow-up may reveal evolving trajectories or shifts in subtype stability that were beyond the scope of this analysis. A further limitation is that the performance of the simplified classifier within the derivation cohort may be optimistic (i.e. overfitting). However, the classifier was subsequently evaluated in the independent BASEL V cohort, providing an external assessment of generalizability. Overall, the four subtypes identified by the simplified classifier in the BASEL V cohort had similar baseline and biological characteristics, and risks of death, compared with the corresponding LCA-derived subtypes in the LEDA cohort, supporting the robustness of the classification approach, although further validation in additional populations would be valuable.

## Conclusion

In the analysis of two prospective independent observational acute dyspnoea cohorts, an unsupervised learning model identified four distinct acute dyspnoea subtypes, labelled as non-inflammatory, tachycardic, anaemic and hypoxemic. These four patient clusters demonstrated gradients in the levels of biological markers and severity of outcomes. Patients’ assignment to the subtype was significantly associated with 3-month mortality even after adjusting for traditional risk factors. Future prospective studies are required to externally validate these subtypes for a more personalized classification approach to patients with acute dyspnoea.

## Supplementary Material

xvag225_Supplementary_Data

## Declarations

### Data availability

The data underlying this article are not publicly available.

### Pre-registered Clinical Trial Number

None supplied.
