# Supplementary material for: Identifying clinical subtypes in acute dyspnoea patients admitted to the emergency department: a secondary model-based clustering of prospective cohorts
Source: ESC Heart Fail. 2026 Aug 25;13(5):xvag225. doi: 10.1093/eschf/xvag225 (PMC13537452; doi:10.1093/eschf/xvag225)
Supplement: Supplementary file 1 [file xvag225_supplementary_data.docx]

**Supplemental Materials**

**Supplemental Table 1.** Measured biomarkers and their function.

| **Biomarker** | **Host response/pathway** | **Assay** |
| --- | --- | --- |
| GDF-15^*,†^ | Cellular stress/inflammation | Roche |
| NT-proBNP^*,†^ | Cardiac stretch/stress | Roche |
| Hs-TnT^*,†^ | Cardiac cell necrosis | Roche |
| Bio-ADM^*,†^ | Endothelial dysfunction | SphingoTec |
| penKid^*^ | Kidney injury | SphingoTec |
| DDP3^*^ | Acute cardiac stress | SphingoTec |
| IL6^†^ | Inflammation | Singulex |
| Ferritin^†^ | Inflammation, iron status | Roche |

^*^ - measured in LEDA cohort;

^†^ - measured in BASEL V cohort.

**Abbreviations:** Bio-ADM – bioactive adrenomedullin; DDP3 – dipeptidyl peptidase 3; GDF-15 – growth differentiation factor 15; hs-TnT – high-sensitivity troponin T; IL-6 – interleukin 6; NT-proBNP – N-terminal pro–B-type natriuretic peptide.

**Supplemental Table 2.** Description of the selection process of the class-defining variables in the LEDA and BASEL V cohorts.

| **Steps (chronological order)** | **Description** |
| --- | --- |
| **I** | Readily available laboratory data and host response biomarkers which were collected/available in both LEDA and BASEL V cohorts were identified by SS. |
| **II** | Different laboratory data and host response biomarkers were preselected based on the prior published literature, their association with organ injury and outcome with a consensus of two reviewers SS and KČB. |
| **III** | SS excluded the variables with more than 25% missing values in at least one of the cohorts. |
| **IV** | The final selection of the nine class-defining biomarker variables was made by consensus among three experts SS, JČ and AM (conference call discussion). |
| **V** | SS assessed the correlation of the nine class-defining variables for an *a priori* value of the Spearman’s rank correlation coefficients lrl <0.5 (Supplemental Figures 2A and 2B). |

**Supplemental Table 3.** Missing data of the variables used in latent class analysis in the derivation (LEDA, n = 1392) cohort.

| **Variable** | **Missing data, n (%)** |
| --- | --- |
| Systolic blood pressure | 5 (<1%) |
| Heart rate | 5 (<1%) |
| SpO2 | 350 (25%) |
| Sodium | 155 (11%) |
| Potassium | 16 (1%) |
| Creatinine | 51 (4%) |
| CRP | 189 (14%) |
| Hemoglobin | 30 (2%) |
| Blood glycemia | 333 (24%) |

**Abbreviations:** CRP – C-reactive protein; SpO₂ – peripheral capillary oxygen saturation.

**Supplemental Table 4**. Availability of class-defining variables in the derivation (LEDA) cohort.

|  | N | % | Cumulative % |
| --- | --- | --- | --- |
| All 9 variables available | 642 | 46% | 46% |
| 8 variables available | 477 | 34% | 80% |
| 7 variables available | 178 | 13% | 93% |
| 6 variables available | 79 | 6% | 99% |
| 5 variables available | 16 | 1% | 100% |
| < 5 variables available | 0 | 0% |  |

**Supplemental Table 5.** Overall baseline characteristics of the derivation (LEDA) and validation (BASEL V) cohorts

| **Characteristic** | **LEDA (Derivation cohort) (n = 1392)** | **BASEL V (Validation cohort) (n = 1886)** |
| --- | --- | --- |
| Age, years | 71 [62–79] | 75 [63–82] |
| Female sex, n (%) | 601 (43.2%) | 833 (44.2%) |
| Acute heart failure, n (%) | 739 (53%) | 975 (52%) |
| Chronic heart failure, n (%) | 857 (61.6%) | 617 (33%) |
| Diabetes mellitus, n (%) | 318 (22.8%) | 446 (23.7%) |
| Chronic kidney disease, n (%) | 248 (17.8%) | 551 (29.2%) |
| Active/recent cancer, n (%) | 197 (14.1%) | 76 (4.0%) |
| 3-month all-cause mortality, n (%) | 194 (13.9%) | 204 (10.8%) |

**Supplemental Table 6. Cox proportional hazards models to adjust for confounding including individual variables used in the LCA** **for 3-month mortality in the LEDA and the BASEL V cohorts.**

**Model A covariates (LEDA cohort):** tertiles of systolic blood pressure, tertiles of SpO2, tertiles of CRP, age (per 10 years), sex, history of CHF, history of diabetes, history of cancer, history of COPD, history of CKD, diagnosis at admission, and hospitalization.

**Model A covariates (BASEL V cohort):** tertiles of systolic blood pressure, tertiles of SpO2, tertiles of CRP, age (per 10 years), sex, history of CHF, history of diabetes, history of cancer, history of COPD/asthma, history of CKD, other diagnosis at admission, AHF at admission, infection precipitating AHF, COPD or asthma at admission, cancer at admission, pulmonary embolism at admission, Afib/flutter precipitating AHF, ACS and AHF at admission.

**Model B covariates (LEDA and BASEL V cohorts):** Subtypes in addition to model A covariates.

| **LEDA Cohort** | **C-index (95% CI)** | **p-value** |
| --- | --- | --- |
| **Model A** | 0.73 (0.72, 0.74) | Referent |
| **Model B** | 0.77 (0.76, 0.78) | 0.04 |

| **BASEL V Cohort** | **C-index (95% CI)** | **p-value** |
| --- | --- | --- |
| **Model A** | 0.76 (0.75, 0.77) | Referent |
| **Model B** | 0.79 (0.78, 0.80) | 0.03 |

Inference was combined from the sets of 20 imputed samples using Rubin’s rules. A significant improvement in Cox regression model discrimination was found when adding subtype membership in model B on top of tertiles of systolic blood pressure, SpO2 and CRP and other confounders in model A with an increase in Harrell’s C-index by 4% (p = 0.04) and 3% (p = 0.03) respectively in the LEDA and the BASEL V cohorts.

Abbreviations:

AHF – acute heart failure; Afib – atrial fibrillation; ACS – acute coronary syndrome; CHF – chronic heart failure; CI – confidence interval; CKD – chronic kidney disease; COPD – chronic obstructive pulmonary disease; CRP – C-reactive protein; HR – hazard ratio; SpO₂ – peripheral capillary oxygen saturation.

**Supplemental Table 7. Comparison of LCA models with different numbers of classes in the LEDA cohort (N=1392).**

| **Class number** | **BIC** | **Entropy** | **n1** | **n2** | **n3** | **n4** | **n5** | **n6** |
| --- | --- | --- | --- | --- | --- | --- | --- | --- |
| 1 | 32439 | – | 1392 | – | – | – | – | – |
| 2 | 31005 | 0.68 | 929 | 463 | – | – | – | – |
| 3 | 30762 | 0.63 | 255 | 779 | 358 | – | – | – |
| 4* | 30583 | 0.63 | 499 | 420 | 348 | 125 | – | – |
| 5 | 30583 | 0.60 | 340 | 91 | 145 | 368 | 448 | – |
| 6 | 30584 | 0.60 | 94 | 315 | 340 | 80 | 126 | 437 |

Bayesian information criterion (BIC) is a metric for model selection; lower values suggest model parsimony. Entropy is a measure of separation between latent classes; higher entropy denotes better class separation. Columns n1–n6 indicate the class sizes for each n-class solution. *Selected four-class LCA model (lowest BIC).

The median [IQR] posterior class-membership probability was 0.84 [0.75–0.96] in the four-class solution.

Abbreviations: LCA – latent class analysis; BIC – Bayesian information criterion; IQR – interquartile range.

**Supplemental Table 8. Comparison of LCA models with different numbers of classes in the LEDA internal derivation cohort (N=835), defined as the first 60% of patients admitted in chronological order.**

| **Class number** | **BIC** | **Entropy** | **n1** | **n2** | **n3** | **n4** | **n5** | **n6** |
| --- | --- | --- | --- | --- | --- | --- | --- | --- |
| 1 | 19392 | – | 835 | – | – | – | – | – |
| 2 | 18558 | 0.70 | 257 | 578 | – | – | – | – |
| 3 | 18447 | 0.64 | 375 | 77 | 383 | – | – | – |
| 4* | 18407 | 0.64 | 207 | 35 | 155 | 438 | – | – |
| 5 | 18417 | 0.60 | 68 | 353 | 201 | 99 | 114 | – |
| 6 | 18443 | 0.63 | 83 | 104 | 168 | 372 | 32 | 76 |

Bayesian information criterion (BIC) is a metric for model selection; lower values suggest model parsimony. Entropy is a measure of separation between latent classes; higher entropy denotes better class separation. Columns n1–n6 indicate the class sizes for each n-class solution. *Selected four-class LCA model (lowest BIC).

The median [IQR] posterior class-membership probability was 0.88 [0.77–0.97] in the four-class solution.

Abbreviations: LCA – latent class analysis; BIC – Bayesian information criterion; IQR – interquartile range.

**Supplemental Table 9. Class-defining variables in the LEDA internal derivation cohort (N=835), first 60% of patients admitted in chronological order.**

| **Variable** | **Subtype A (non-inflammatory)** | **Subtype B (tachycardic)** | **Subtype C (anaemic)** | **Subtype D (hypoxemic)** | **p value** |
| --- | --- | --- | --- | --- | --- |
|  | (n=438) | (n=207) | (n=155) | (n=35) |  |
| Systolic blood pressure, mmHg | 140 [130, 160] | 146 [127, 170] | 120 [107, 140] | 122 [110, 139] | <0.001 |
| Heart rate, bpm | 84 [72, 100] | 94 [78, 115] | 84 [72, 100] | 109 [91, 125] | <0.001 |
| SpO2, % | 95 [93, 97] | 90 [86, 94] | 92 [90, 95] | 73 [65, 78] | <0.001 |
| Sodium, mmol/L | 140 [138, 142] | 137 [135, 140] | 135 [130, 138] | 137 [136, 139] | <0.001 |
| Potassium, mmol/L | 4.2 [3.9, 4.5] | 4.2 [3.8, 4.6] | 4.4 [4.0, 4.7] | 4.6 [4.2, 4.9] | <0.001 |
| Creatinine, µmol/L | 85 [73, 105] | 106 [82, 137] | 122 [84, 176] | 105 [83, 134] | <0.001 |
| CRP, mg/L | 5.7 [2.3, 12.0] | 16.6 [5.5, 40.8] | 37.1 [11.7, 91.2] | 39.5 [15.8, 71.0] | <0.001 |
| Haemoglobin, g/L | 13.6 [12.6, 14.6] | 13.1 [11.9, 14.5] | 10.7 [8.9, 12.6] | 12.6 [10.5, 14.9] | <0.001 |
| Blood glycaemia, mmol/L | 5.7 [5.3, 6.4] | 9.2 [7.0, 11.4] | 6.4 [5.6, 7.2] | 6.7 [6.2, 7.3] | <0.001 |

Data are presented as medians [IQR]. Comparisons across all four subtypes were performed using the Kruskal–Wallis test.

Abbreviations: CRP – C-reactive protein; IQR – interquartile range; SpO2 – peripheral capillary oxygen saturation.

**Supplemental Table 10. Comparison of LCA models with different numbers of classes in the LEDA internal validation cohort (N=557), defined as the last 40% of patients admitted in chronological order.**

| **Class number** | **BIC** | **Entropy** | **n1** | **n2** | **n3** | **n4** | **n5** | **n6** |
| --- | --- | --- | --- | --- | --- | --- | --- | --- |
| 1 | 13128 | – | 557 | – | – | – | – | – |
| 2 | 12595 | 0.68 | 322 | 235 | – | – | – | – |
| 3 | 12530 | 0.66 | 188 | 282 | 87 | – | – | – |
| 4* | 12503 | 0.66 | 71 | 192 | 170 | 124 | – | – |
| 5 | 12529 | 0.65 | 135 | 141 | 31 | 206 | 44 | – |
| 6 | 12568 | 0.66 | 117 | 52 | 174 | 40 | 70 | 104 |

Bayesian information criterion (BIC) is a metric for model selection; lower values suggest model parsimony. Entropy is a measure of separation between latent classes; higher entropy denotes better class separation. Columns n1–n6 indicate the class sizes for each n-class solution. *Selected four-class LCA model (lowest BIC).

The median [IQR] posterior class-membership probability was 0.88 [0.79–0.96] in the four-class solution.

Abbreviations: LCA – latent class analysis; BIC – Bayesian information criterion; IQR – interquartile range.

**Supplemental Table 11. Class-defining variables in the LEDA internal validation cohort (N=557), last 40% of patients admitted in chronological order.**

| **Variable** | **Subtype A (non-inflammatory)** | **Subtype B (tachycardic)** | **Subtype C (anaemic)** | **Subtype D (hypoxemic)** | **p value** |
| --- | --- | --- | --- | --- | --- |
|  | (n=192) | (n=124) | (n=170) | (n=71) |  |
| Systolic blood pressure, mmHg | 140 [129, 160] | 153 [138, 165] | 130 [117, 145] | 139 [117, 164] | <0.001 |
| Heart rate, bpm | 80 [70, 100] | 103 [88, 120] | 80 [71, 94] | 92 [74, 110] | <0.001 |
| SpO2, % | 96 [95, 97] | 89 [86, 91] | 95 [92, 97] | 86 [78, 94] | <0.001 |
| Sodium, mmol/L | 141 [140, 142] | 138 [136, 140] | 138 [135, 141] | 134 [127, 140] | <0.001 |
| Potassium, mmol/L | 4.3 [4.0, 4.6] | 4.4 [3.9, 4.6] | 4.4 [4.0, 4.8] | 4.6 [4.1, 5.1] | 0.003 |
| Creatinine, µmol/L | 82 [71, 94] | 94 [75, 116] | 116 [78, 157] | 111 [71, 241] | <0.001 |
| CRP, mg/L | 4.0 [1.3, 9.3] | 19.8 [8.6, 39.5] | 18.3 [4.7, 52.1] | 26.3 [6.2, 71.7] | <0.001 |
| Haemoglobin, g/L | 13.5 [12.6, 14.4] | 13.9 [12.8, 14.8] | 11.3 [9.8, 13.0] | 12.0 [10.6, 14.1] | <0.001 |
| Blood glycaemia, mmol/L | 5.7 [5.3, 6.7] | 7.5 [6.0, 9.6] | 6.2 [5.4, 7.0] | 6.7 [5.4, 9.7] | <0.001 |

Data are presented as medians [IQR]. Comparisons across all four subtypes were performed using the Kruskal–Wallis test.

Abbreviations: CRP – C-reactive protein; IQR – interquartile range; SpO2 – peripheral capillary oxygen saturation.

**Supplemental Figure 1.** A simplified classifier (decision tree) of the derivation LEDA cohort for subtype assignment in the validation cohort.

CRP – C reactive protein; SpO2 – peripheral oxygen saturation.

| Subtype | Sensitivity (95% CI) | Specificity (95% CI) |
| --- | --- | --- |
| D (vs C+B+A) | 64% (51-76) | 99% (98-99.5) |
| C (vs D+B+A) | 71% (63-77) | 93% (91-95) |
| B (vs D+C+A) | 73% (66-79) | 88% (85-91) |
| A (vs D+C+B) | 91% (86-94) | 87% (84-90) |

Ability of the simplified classifier to discriminate LCA-derived subtypes in the LEDA cohort patients with all available data (642 patients). The simplified classifier demonstrated good performance in distinguishing the four LCA classes in the LEDA cohort, yielding a Hand–Till multiclass AUC (95% CI) of 0.87 (0.85–0.88) and a Brier score of 0.19, indicating low overall prediction error. The classification rates obtained using the simplified classifier are presented at the bottom of the figure; for each subtype, a single box displays four successive classification rates corresponding to subtypes A, B, C and D, respectively. Abbreviations: AUC – area under the curve; CI – confidence interval; CRP – C-reactive protein; SpO2 – peripheral oxygen saturation.

**Supplemental Figure 2A.** Heatmap of correlation between the variables used in latent class analysis in the derivation (LEDA) cohort. Heatmap shows greater color (red or blue) when the Spearman’s rank correlation coefficient is greater in positive or negative direction.

**Abbreviations:** CRP – C reactive protein; SpO2 – peripheral oxygen saturation.

**Supplemental Figure 2B.** Heatmap of correlation between the variables used in latent class analysis in the validation (BASEL V) cohort. Heatmap shows greater color (red or blue) when the Spearman’s rank correlation coefficient is greater in positive or negative direction.

**Abbreviations:** CRP – C reactive protein; SpO2 – peripheral oxygen saturation.

**Supplemental Figure 3.** Flowchart of the BASEL V patient cohort.

**Supplemental Figure 4.** BIC curve of LCA analysis with k=1-8

**Supplemental Figure 5.** 3-month cardiovascular and non-cardiovascular mortality in acute dyspnoea cohorts based on the 4 clusters.

**Subtype labels:** A – ‘non-inflammatory’, B – ‘tachycardic’; C – ‘anaemic’ and D – ‘hypoxemic’.

A. Cardiovascular mortality in the derivation (LEDA) cohort.
B. Non-cardiovascular mortality in the derivation (LEDA) cohort.
C. Cardiovascular mortality in the validation (BASEL V) cohort.
D. Non-cardiovascular mortality in the validation (BASEL V) cohort.

**Supplemental Figure 6.** 3-month all-cause, cardiovascular and non-cardiovascular readmission based on 4 clusters of the derivation (LEDA) cohort.

**Subtype labels:** A – ‘non-inflammatory’, B – ‘tachycardic’; C – ‘anaemic’ and D – ‘hypoxemic’.

**Abbreviations:** CV – cardiovascular.

**Supplemental Figure 7.** 3-month all-cause, cardiovascular and non-cardiovascular mortality in acute heart failure patients based on 4 clusters of the derivation (LEDA) cohort.

**Subtype labels:** A – ‘non-inflammatory’, B – ‘tachycardic’; C – ‘anaemic’ and D – ‘hypoxemic’.

**Abbreviations:** AHF – acute heart failure.

**Supplemental Figure 8.** 3-month all-cause, cardiovascular and non-cardiovascular mortality in non-acute heart failure patients based on 4 clusters of the derivation (LEDA) cohort.

**Subtype labels:** A – ‘non-inflammatory’, B – ‘tachycardic’; C – ‘anaemic’ and D – ‘hypoxemic’.

**Abbreviations:** AHF – acute heart failure.

**Supplemental Figure 9.** 3-month all-cause readmission or mortality in acute dyspnoea cohorts based on the four subtypes in **(A)** the derivation (LEDA) cohort and **(B)** the validation (BASEL V) cohort.

**Subtype labels:** A – ‘non-inflammatory’, B – ‘tachycardic’; C – ‘anaemic’ and D – ‘hypoxemic’.

**Supplemental Figure 10**. Radar (spider) plot of the average standardised clinical and biological profiles across the four LCA-derived subtypes in the LEDA cohort.

**Supplemental Figure 11.** Comparison of class-defining variables using latent class analysis at inclusion in the LEDA internal derivation cohort (N=835), defined as the first 60% of patients admitted in chronological order.

Description: continuous variables were plotted after natural-log transformation; every variable was standardised so that means are scaled to 0 and SDs to 1. Group means of standardised values are shown by subtype. A value of +1 for a standardised variable (y-axis) indicates that the mean value for a given subtype was one SD higher than the mean value in the whole cohort. Subtype labels: A – 'non-inflammatory', B – 'tachycardic', C – 'anaemic' and D – 'hypoxemic'. Abbreviations: SD – standard deviation; CRP – C-reactive protein; SpO2 – peripheral oxygen saturation.

**Supplemental Figure 12.** Comparison of class-defining variables using latent class analysis at inclusion in the LEDA internal validation cohort (N=557), defined as the last 40% of patients admitted in chronological order.

Description: continuous variables were plotted after natural-log transformation; every variable was standardised so that means are scaled to 0 and SDs to 1. Group means of standardised values are shown by subtype. A value of +1 for a standardised variable (y-axis) indicates that the mean value for a given subtype was one SD higher than the mean value in the whole cohort. Subtype labels: A – 'non-inflammatory', B – 'tachycardic', C – 'anaemic' and D – 'hypoxemic'. Abbreviations: SD – standard deviation; CRP – C-reactive protein; SpO2 – peripheral oxygen saturation.

**Supplemental Figure 13.** Comparison of class-defining variables using the simplified classifier at inclusion in the LEDA cohort (N=1392).

Description: continuous variables were plotted after natural-log transformation; every variable was standardised so that means are scaled to 0 and SDs to 1. Group means of standardised values are shown by subtype. A value of +1 for a standardised variable (y-axis) indicates that the mean value for a given subtype was one SD higher than the mean value in the whole cohort. Subtype labels: A – 'non-inflammatory', B – 'tachycardic', C – 'anaemic' and D – 'hypoxemic'. Abbreviations: SD – standard deviation; CRP – C-reactive protein; SpO2 – peripheral oxygen saturation.
